# Birth preparedness and complication readiness practice among women attending antenatal care follow up in Yirgalem general hospital, southern Ethiopia

**DOI:** 10.1371/journal.pgph.0000864

**Published:** 2022-08-05

**Authors:** Molalegn Mesele, Walellign Anmut

**Affiliations:** Department of Midwifery, College of Health Science and Medicine, Wolaita Sodo University, Wolaita Sodo, Ethiopia; The University of Texas Health Science Center at Houston School of Public Health, UNITED STATES

## Abstract

In developing countries, maternal and newborn mortality is a major public health issue. Birth preparedness and complication readiness is a method to encourage pregnant women to seek professional birth attendants as soon as possible. The aim of this study was to evaluate practice and factors associated with birth preparedness and complication readiness among women attending antenatal care, southern Ethiopia, in 2019. From September 1st to September 30th, 2019, a facility-based cross-sectional study was conducted. 422 pregnant women were randomly selected and interviewed using a structured questionnaire. Epi-data version 3.1 was used to enter data, while SPSS version 21 was used to analyze it. To find factors associated with birth preparedness and complications readiness, researchers used multivariable logistic regression.From 422 study participants, 205(48.6%) (95% CI: 46.9%, 49.8%) have birth preparedness and complication readiness practice. Age of respondent ≥ 37 years (AOR = 4.2, 95% C.I = 1.23, 14.24) and between 25 to 30 (AOR = 2.35, 95% C.I = 1.1, 5.1); level of education College and above(AOR = 5.59, 95% C.I 2.8, 11.2) and secondary school (AOR = 9.5, 95% C.I 3.99–22); previous history of ANC follow up (AOR = 4.33, 95% C.I = 2.46, 7.61), birth outcome with live birth(AOR = 3.53, 95% C.I = 1.51, 8.25), and history of birth at health facility (AOR = 3.09, 95% C.I = 1.72, 5.56) where factors significantly associated with birth preparedness and complication readiness practice. Overall, there was low birth preparedness and complication readiness practices were observed in current study. Age of respondents, level of education, history of ANC follow up, and history of birth at a health facility were factors associated with birth preparedness and complication readiness practice. Governments with other stakeholders should work by focusing on antenatal care and institutional delivery by focusing on older age group mothers with who has no formal education.

## Introduction

More than 40% of pregnant women worldwide are at risk of developing serious obstetric problems. According to the World Health Organization (WHO), more than 300 million women in developing countries suffer from short- and long-term problems connected to pregnancy and childbirth [1]. The global Maternal Mortality Ratio (MMR) decreased from 385 deaths per 100,000 live births in 1990 to 216 in 2015, according to data from 171 countries, with 303,000 maternal deaths globally in 2015. This is composed of 99% women from developing countries [2]. Birth preparations and complication readiness is a method that encourages pregnant women, their families, and individuals to plan for childbirth and deal with any issues that may arise. It is an important component of internationally recognized safe motherhood initiatives [3].

For the purpose of promoting quick access to skilled maternal health care, the ideas of birth preparation and complication readiness have been integrated. In the event of pregnancy or childbirth complications, it helps expectant mothers and their relatives to get immediate medical care. The processes of preparing for a typical birth while also foreseeing the actions that will be necessary in the event of an emergency are referred to as birth preparedness and complication readiness [4]. Because childbirth is an unknown risk factor, giving prompt and adequate health care for pregnant women who are experiencing obstetric complications may be a viable option for justifying the risk [5].

The essential measures for timely utilization of skilled maternal and neonatal care, especially during pregnancy, labor, and childbirth, include identifying and decreasing delays in seeking, reaching, and acquiring care [6]. One of the most important aspects of a globally accepted safe motherhood program is delivery readiness and difficulties. The term "readiness" refers to the availability of maternal health care services throughout pregnancy as well as the risk for difficulties [5, 7].

All through Antenatal care visits in Ethiopian Healthcare Facilities and all levels of hospitals, women were given information about specific pregnancy complications such as a severe headache, abdominal pain, vaginal bleeding, vaginal fluid leakage, and blurred vision, but only half of pregnant women in developing countries receive the suggested minimum of 4 ANC care visits [8].

Birth preparedness and complication readiness are two of the main areas of involvement, with the goal of reducing neonatal and maternal mortality by promoting health-seeking behavior and the use of appropriate skilled people and health-care facilities for delivery [9]. The birth readiness and complication readiness matrix was created by the maternal and neonatal health program of the Johns Hopkins Program for International Education in Gynecology and Obstetrics (JHIEGO) to address these three delays: (A) the delay in deciding to seek care for an obstetric complication; (B) the delay in traveling to a health facility once the decision to seek care has been made; and (C) the delay in receiving care once at the facility [10]. From study done in Ethiopia; age, Antenatal Care (ANC) follow up, place of residence, educational status, birth at health facility, history of still birth and knowledge of birth preparedness and complication readiness plan are factors associated with birth preparedness and complication readiness practice [11, 12].

Also, little was known about birth preparedness and complication readiness practice and factors associated with birth preparedness and complication readiness in the study area and the region as well. Therefore, this study aimed to fill the gap by assessing the current status and factors associated with birth preparedness and complication readiness practice among women attending ANC in Yirgalem Hospital, southern Ethiopia.

## Method and material

### Ethics statement

Ethical approval was obtained from Wolaita Sodo University College of health science and medicine Ethical review board. Permission was obtained from Sodo town health office.

#### Consent to participate

Oral informed consent was obtained from the parents of the minors and study participants. The purpose and objective of the study was well explained to study participants and they were informed about their full right to withdrawal or discontinue participation at any time they want. In addition privacy and confidentiality of participant was kept.

### Study setting and design

Health facility based cross sectional study was conducted in Yirgalem general Hospital from September 1st to 30th, 2019. Yirgalem general Hospital is located in Sidama zone, Southern Nation Nationality People Republic (SNNPR), Ethiopia. The Hospital has a catchment population of 4.6 million and 260 births per month. Figures from the central statistics agency of Ethiopia published in 2005 indicated that. Yirgalem has an estimated total population of 43,815 of whom 21,975 women and 21,840 are men. Yirgalem town is located at the distance of 260Km from Addis Abeba the capital city of Ethiopia and 40Km from Hawassa the capital city of SNNPR. The study was carried out among pregnant women attending antenatal care.

### Source and study population

All pregnant womens who came to Yirgalem general hospital for ANC visit were the source population and all pregnant women who fulfill the inclusion criteria and selected pregnant women were a study population.

### Eligibility criteria

Pregnant women who can hear and communicate and who are volunteering to participate are included and a pregnant women with mental problem or seriously ill/ unable to hear and speak and primigravida women were excluded.

### Sample size determination and sampling procedure

The sample size was estimated by using sample size determination for a single population proportion formula. Therefore, the total sample size was calculated with the marginal error of 0.05, with 95% confidence interval and p-value 48.5% from a study conducted in Sodo town, Wolaita Zone, SNNPR, Ethiopia [11].

n = sample size

Z = standard error with the given level of confidence

D = margin of error 5%

Confidence interval 95%

P = 48.5%

N = Z (a/2)2 p (1-p)

                d2

        = (1.96)2 0.485(1–0.485)

                    (0.05)2

                  N = 383

A possibility of 10% of the total sample size was considered for non-respondents. And the final sample size was 422.

### Sampling technique

A systematic random sampling technique was used to select 422 pregnant women who attend ANC in Yirgalem Hospital. The first women was selected by simple random sampling; lottery method. Then the next women was selected through systematic sampling technique every 5th interval from the order of mothers who come for ANC; until the desired numbers of the sample were obtained.

### Data collection procedure and instrument

Face-to-face interviews were used to collect data, and the questionnaire was structured. The questionnaire, which includes socioeconomic and demographic variables, obstetric, health-care-related aspects, information sources, and transportation access, was initially prepared in English and then translated into Amharic. The Johns Hopkins Initiative for International Education in Gynecology and Obstetrics developed the questionnaire for the mother and child health program. The practice of birth preparedness and complication readiness is the result of this research [10].

### Operational definition

Birth preparedness and complication readiness: A women was considered as prepared for birth and its complication if she save money for birth-related and emergency expense; identified place of delivery; skill birth attendants; arrange transport to health institution for delivery and obstetric urgent situation, and prearranged blood donor in cases of obstetrics emergency. The 5 variables were transformed on SPSS in to one variable that is “birth preparedness and complication readiness practice” women who found at least 3 of the 5 birth preparedness and complication readiness component were considered as” prepared for birth and its complication” coded as 1. The remain women who found less than three were considered as “not prepared for birth and its complication” and coded as 0. This scoring has been previously used in studies that assessed women’s birth preparedness and complication readiness practice [11, 13, 14].

### Data processing and analysis

Data were checked for completeness and entered in to Epi data version 3.1 and SPSS window version 21 was used for analysis. Descriptive analysis was done and the results were presented in the form of narrative and table. bivariable and multivariable logistic regression was done to identify factors associated with birth preparedness and complication readiness. All variables with p-value less than 0.25 in bivariable logistic regression model were entered to a multivariable logistic regression model for controlling possible confounding and odds ratios and their 95% CIs were computed.

## Result

### Socio demographics characteristics

During the study, data were collected from 422 pregnant women who visited Yirgalem General Hospital for ANC follow-up. The means with standard deviation (SD) of the age of the respondents was (26.3 ± 6.2). The majority of respondents 252 (59.7%) were between the ages of 25 and 30, with 148 (35.1%) having only an elementary education. The majority of the responders 398, (94.3%) were married (Table 1).

**Table 1:** Socio-demographic characteristics of women attending ANC follow up in Yirgalem general Hospital, Sidama Zone, Ethiopia; 2019 (n = 422).

Variable	Frequency	Percent
**Age of respondents**		
18–24	64	15.2
25–30	252	59.7
31–36	76	18
37–44	30	7.1
**Level of education**		
No formal education	135	32
Primary education	148	35.1
Secondary education	40	9.5
College and university	99	23.5
**Occupation**		
House wife	212	50.2
Farmer	77	18.2
Merchant	78	18.5
Government employee	12	2.8
Private employee	43	10.2
**Estimated monthly imcome (ETB)**		
>500	273	64.7
500–3500	104	24.6
3600–7000	45	10.7
**Current marital status**		
Married	398	94.3
Not married	19	4.5
separated	5	1.2
**Age at first marriage**		
**None**	16	3.8
14–18	252	59.7
19–25	120	28.4
26–32	24	5.7
33–40	10	2.4
**Educational level of husband**		
No formal education	33	7.8
Primary education	138	32.7
Secondary education	71	16.8
College and university	180	42.6
**Occupation of husband**		
No work	21	5
Farmer	144	34.1
Merchant	108	25.6
Government employee	35	8.3
Daily laborer	70	16.6
Self employee	44	10.4
**Estimated monthly income of husband (ETB)**		
>500	75	17.8
500–3500	130	30.8
3600–7000	133	31.5
8000 and above	84	19.9
**Numbers of person live in the house hold**		
1–3	157	37.2
4–5	246	58.3
>5	19	4.5
**Decision maker in the house hold**		
Herself	33	7.8
Husband	86	20.4
Self and husband	294	69.7
Other family	9	2.1

### Obstetrics characteristics of participants

Three hundred seventeen (75.1%) of participants had attended ANC during their previous pregnancy, according to obstetrics characteristics. In terms of pregnancy outcomes, 74 (17.5%) of mothers have had an abortion, 256 (60.6%) have had live births, and only six have had still births. 281 (66.6%) of mothers give delivery in a medical facility (Table 2).

**Table 2:** Obstetrics characteristics of women attending ANC follow up in Yirgalem general Hospital, Sidama Zone, Ethiopia; 2019 (n = 422).

Variable	Frequency	Percent
**Age at first pregnancy**		
<18	81	19.2
18–25	129	30.6
26–36	169	40
36–45	43	10.2
**Outcome face during pregnancy**		
No outcome	86	20.4
Abortion	74	17.5
Live birth	256	60.9
Still birth	6	1.4
**Presence of pregnancy now**		
Yes	412	97.6
No	10	2.4
**Previous ANC follow up**		
Yes	317	75.1
No	105	24.9
**ANC is useful**		
Yes	407	96.4
No	15	3.6
**Birth at health facility**		
Yes	281	66.6
No	141	33.4
**Week to start ANC**		
None	107	25.4
Before 16 week	177	41.9
16–28 week	111	26.3
28–34 week	19	4.5
34–40 week	8	1.9
**Time of attending ANC in last pregnancy**		
None	104	24.6
One	29	6.9
Twice	42	10
Three time	68	16.1
Four and above	178	42.4

### Women’s birth preparedness plan and complication readiness

**Fig 1:**
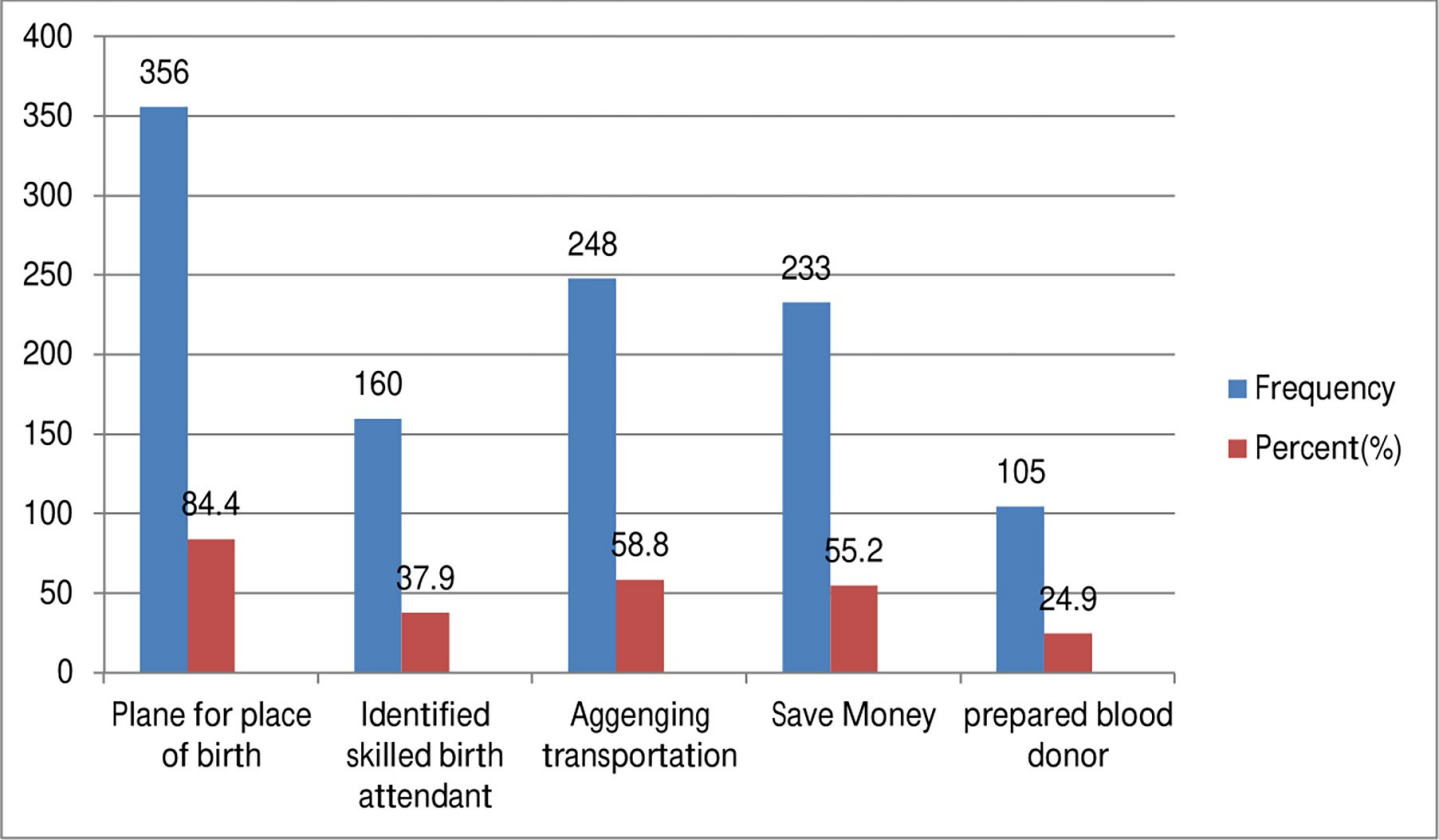
Components of Birth preparedness and complication readiness items in Yirgalem general Hospital, Sidama Zone, Ethiopia; 2019 (n = 422).

The Birth Preparedness and Complication Readiness (BPCR) rate was examining three or more stages from these five components, 205 people (48.6%) (95% CI: 46.9%, 49.8%) were found to meet the criterion and be ready for birth and its complications (Fig 1).

### Factors associated with birth preparedness and complication readiness practice

In a multivariable regression analysis, respondents’ age, level of education, pregnancy and childbirth outcomes, attendance at antenatal care during the previous pregnancy, birth outcome with live birth, and birth at a health facility were all significantly related to birth preparedness and complication readiness practice. Birth preparedness and complication readiness practice is higher in older age groups than in younger age groups; age groups 37–44 are four times more likely to practice birth preparedness and complication readiness (AOR = 4.2, 95% C.I = 1.23, 14.24) than younger age groups, and age groups 25–30 are more than two times more likely to practice birth preparedness and complication readiness (AOR = 2.35, 95% C.I = 1.1, 5.1) than younger age groups.

Another factor that was linked to birth preparations and complication readiness was educational level. When compared to participants with no formal education, those with a college education or more were more than 5 times as likely to practice birth preparedness and complication readiness (AOR = 5.59, 95% C.I 2.8, 11.2), Participants with a secondary school education were more than 9 times (AOR = 9.5, 95% C.I 3.99–22) as likely as those without a formal education to have birth preparedness practice, and mothers with a primary school education were more than 2 times (AOR = 2.8, 95% C.I 1.46–5.38) as likely as those without a formal education to have birth preparedness and complication readiness practice.

History of antenatal care follow up was another significant factor associated with birth preparedness and complication readiness; those who have had previous history of antenatal care follow up are 4.33 times more likely to have birth preparedness and complication readiness practice (AOR = 4.33, 95% C.I = 2.46, 7.61) than those who have not. One of the birth outcomes that was significantly associated with birth preparedness and complication readiness practice was live birth. When compared to mothers who had no delivery outcome, mothers who had a live delivery were more than three times more likely (AOR = 3.53, 95% C.I = 1.51, 8.25) to have had birth preparations and complication readiness practice. Another factor that has been connected to birth preparations and complication preparation practice is giving birth in a health facility. Those who have previously given birth in a health institution are more likely to practice birth preparedness and complication readiness (AOR = 3.09, 95% C.I = 1.72, 5.56) than those who have never given birth in a health facility (Table 3).

**Table 3:** Factors affecting birth preparedness and complication readiness practice in Yirgalem general Hospital, Sidama Zone, Ethiopia; 2019 (n = 422).

Variable	birth preparedness and complication readiness practice		Crude OR (95% C.I)	Adjusted OR (95% C.I)
	No	Yes		
Age of respondents				
18–24	38(58.5)	27(41.5)	1	1
25–30	128(50.8)	124(49.2)	1.36(0.78,2.36)	2.35(1.07, 5.15)*
31–36	43(57.3)	32(42.7)	1.04(0.53,2.05)	0.87(0.32, 2.33)
37–44	8(26.7)	22(73.3)	3.87(1.50,9.98)	4.17(1.22, 14.24)*
Respondents level of education				
No formal education	100(72.5)	38(27.5)	1	1
Primary education	65(54.2)	55(45.8)	2.22(1.32,3.73)	2.80(1.46, 5.38)*
Secondary education	12(24)	38(76)	8.33(3.94,17.62)	9.52(3.99, 22.68)*
College and university	40(35.1)	74(64.9)	4.862.84,8.32)	5.59(2.79, 11.20)*
Husband occupation				
Farmer	65(30)	79(38.5)	2.0(0.9,4.4)	0.33(0.09, 1.15)
Merchant	60(27.6)	48(23.4)	0.7(0.3, 1.49)	1.01(0.40, 2.84)
Government employed	11(5.1)	24(11.7)	2.6(0.6,10.9)	0.92(0.37, 2.23)
Daily laborer	45(20.7)	25(12.2)	1	1
**Estimated monthly imcome (ETB)**				
>500	122	44.3	0.7(0.37, 1.33)	1.2(0.87, 6.02)
500–3500	59	56.7	1.1(0.56, 2.31	0.6(0.4, 2.1)
3600–7000	24	53.3	**1**	1
Outcome face during pregnancy				
None	53(61.6)	33(38.4)	1	1
Abortion	35(47.3)	39(52.7)	0.91(0.49,1.9)	1.33(0.55, 3.20)
Live birth	82(46.1)	94(53.9)	1.83(0.86,3.1)	3.53(1.51, 8.25)*
Still birth	0(0)	5(100)	1.72(1.01,2.94)	1.55(0.73, 3.28)
Other	47(59.5)	32(40.5)	2.23(1.36,7.32)	
Age at first pregnancy				
<18	45(55.6)	36(44.4)	2.16(1.7,6.67)	1.76(0.44, 7.00)
18–25	62(48.1)	67(51.9)	1.63(0.53,4.98)	1.01(0.26, 3.80)
26–36	93(55)	76(45)	6.00(1.52,23.6)	5.67(0.84, 28)
36–45	17(39.53)	26(60.47)	1	1
Previous ANC follow up				
Yes	150(48.1)	162(51.9)	3.86(1.08,2.62)	4.33(2.46, 7.61)*
No	67(60.9)	43(39.9)	1	1
Birth at health facility				
Yes	110(41.8)	153(58.2)	2.83(1.87,4.28)	3.09(1.7, 5.56)*
No	106(67.1	52(32.9)	1	

*Significant association at P-value <0.05

## Discussion

Nonetheless, birth readiness and complication readiness are two proven and effective health-care strategies for reducing maternal and child mortality, particularly in countries with a high risk of maternal death and an inadequate health-care system. This study was conducted to assess birth preparedness and complication readiness practice and associated factors among mothers attending ANC in Yirgalem hospital. The overall prevalence of BPCR practice was 48.6%. The findings of this study is consistent with those of a study conducted in Wolaita Sodo, a town in southern Ethiopia, which found 48.5% [11] and in rural community in southern Nigeria 48.4% [15]. The similarity may be due to similarity in methodology and socio-demographic characteristics.

In comparison to a study conducted in Jimma Zone, south west Ethiopia (23.3%) [16], Dale District, southern Ethiopia (22.5%) [17], Adigrat town, North Ethiopia (22%) [18], South west Ethiopia (42.3%) [19], and a study conducted in Goba, East Ethiopia (29.9%) [20]. It’s possible that the discrepancy relates to the passage of time. The current study was carried out following extensive multi-sectoral collaboration to improve mother birth preparedness and complication preparation through one-to-five networks health extension workers. And, when compared to a study conducted in Bangkok, Thailand (78.4%) [21] and a study conducted in India (78.4%), the current study is lower [22]. The difference could be due to socio-demographic and socio-economic difference.

Plan for place of birth 84.4%, identify experienced birth attendants 37.9%, arrange transportation 58.8%, save money 55.2%, and prepared blood donor 24.9% is the most common BPCR practice indications. This finding is nearly similar with a study done in Bench Maji Zone, Southwest Ethiopia 83.7% of mothers identify place of birth, 67.4% identifying skilled birth attendants, 45.2% saving money, 16.8% identification mode of transport and 5.6% arrange blood donor [19]. A study done in Bangkok, Thailand showed that, plan to give birth with skilled provider 92.5%, plan to save money for a child birth 42.5% and plan to identify a mode of transport to place of birth 91% [16]. According to a study conducted in Sodo town, planes for place of birth are used 69.9% of the time, skilled birth attendants are identified 42.6% of the time, transportation is arranged 38.4% of the time, blood donors are prepared 1.6% of the time, and money is saved 86.9% of the time [11].

Birth preparedness and complication readiness are more common in the older age groups than in the younger age groups; the age group 37 was four times more likely to practice birth preparedness and complication readiness than the age group 18–24. The age group 25 to 30 was more than two times more likely to practice birth preparedness and complication readiness than the age group 18–24. The findings are consistent with research conducted in India among women who were beneficiaries of a rural primary health facility in the Dakshina district of Karnataka ^17^. The reason for this could be that younger women have never had an obstetrics issue, therefore they do not need to prepare, whereas older women who have had an obstetrics issue in the past do need to prepare for their current pregnancy (not assessed in the study). This The findings contrast with those of a study conducted in Wolaita Zone, Sodo town, which found that younger age groups are more likely to practice birth readiness and complication readiness [11]. The observed difference could be due to the probably older women in Sodo town are more likely to be conventional and accept because they feel safe delivering at home, and older women are less likely to be birth preparedness and complication readiness.

Educational level was another significantly associated with birth preparedness and complication readiness practice. From the respondents those whose educational level college, University and above are more than 5 time, educational level with secondary school more than 9 time and primary school education more than 2.8 time more likely to have birth preparedness and complication readiness practice than no formal education. The finding of this study was in line with the study done in Wolaita zone, Sodo town, Southern Ethiopia and Robe Woreda, Arisi zone, Central Ethiopia [11, 16]. The reason might be education gives a greater health care seeking behaviors of women and education build self-confidence and decision making ability that enhance their birth preparedness and complication readiness.

Birth preparedness and complication readiness practice are positively associated with antenatal care follow-up history; those who have had antenatal care follow-up in the past are 4.33 times more likely to have birth preparedness and complication readiness practice than those who have never had antenatal care follow-up. This research is in accord with research conducted in Adigrat, Ethiopia, and Wolaita Zone, Sodo, Ethiopia [11, 17]. The reason for this could be that during ANC, the health professional provides birth preparedness and complication readiness education, and the women’s previous exposure will strengthen their knowledge and practice.

Live birth was one of the birth outcomes that was found to be substantially linked to birth preparedness and complication readiness practice. Mothers who had a live delivery were more than three times more likely to have undergone birth preparations and complication readiness practice than mothers who did not have a live delivery. When compared to those who have never given birth in a health facility, those who have a history of giving birth in a health facility are 3.09 times more likely to have birth preparedness and complication readiness practice. This study’s findings are comparable to those of a research conducted in the Dale District in Southern Ethiopia [17]. The reason might be their contact with health institution helps the women to get health education for birth preparedness and complication readiness practice.

The current study’s strengths were the high sample size, well-trained research assistants who interviewed the participants, and the BPCR questionnaire, which was adapted from JHPIEGO. However, there are certain limitations to the study. First, it is an institution-based study that only addresses women who visit a health facility; women who do not visit a health facility miss out on assessing their birth preparedness and complication readiness. The study’s cross-sectional design may make it difficult to discern a temporal relationship between the dependent and independent variables.

## Conclusion

Overall, there was low level of birth preparedness and compilation complication readiness practice. Birth preparedness and complication readiness were found to be strongly correlated with respondents’ age, level of education, age at first pregnancy, outcome face during pregnancy and childbirth, previous antenatal care follow up, and birth at a health institution.

The government has to work in collaboration with different stockholder on institutional delivery coverage with health care provider expected to do not miss the opportunity to disseminate all information related BPCR practice.

## Supporting information

S1 Text (DOCX)

## Abbreviations

AOR: Adjusted Odd Ratio
ANC: Ante Natal Care
BPCR: Birth Preparedness Complication Readiness
ETB: Ethiopian birr
JHIEGO: Johns Hopkins Program for Institutional Education in Gynecology and Obstetrics
SNNPR: Southern Nation Nationality People Republic
