# Supplementary material for: Birth preparedness and complication readiness practice among women attending antenatal care follow up in Yirgalem general hospital, southern Ethiopia
Source: PLOS Glob Public Health. 2022 Aug 5;2(8):e0000864. doi: 10.1371/journal.pgph.0000864 (PMC10021876; doi:10.1371/journal.pgph.0000864)
Supplement: S1 Text — (DOCX) [file pgph.0000864.s001.docx]

Annex –II English version structured questionnaire.

PART-I Socio -demographic Information

| S.No | Question | Respondents possible answer | Code | Skip |
| --- | --- | --- | --- | --- |
| 101 | How old are you (in completed year)? | -------------years |  |  |
| 102 | What is your level of education? | 1-Illitrate  2-can read and write  3-Grade1-8  4-secondary and above |  |  |
| 103 | What is your occupation? | 1-House wife  2-Farmer  3-Marchant  4-House maid  5-Government employee 6-Others specify----------- |  |  |
| 104 | How much do you earn per month ETB? | ---------------------ETB |  |  |
| 105 | What is your current marital status? | 1-Married  2-Never married  3-Seprate  4-Divorced  5-Widowed |  |  |
| 106 | What was your age at first marriage | -----------------years |  |  |
| 107 | What is the level of education of your husband (for those married only)? | 1-Illitrate  2-Can read and write  3-1-8 grade  4-Secondary and above |  |  |
| 108 | Occupation of your husband(for those married only) | 1-Farmer  2-Marchant  3-Government employee 4-Daily laborer  5-other specify------------ |  |  |
| 109 | How much income does your husband get from his work /month | --------------in ETB  --------------in quality |  |  |
| 110 | How many of you are living in your house hold | -------------- |  |  |
| 111 | Who is the decision maker in the house hold | 1-Self  2-Husband  3-Self and husband  4-Others,specify----------- |  |  |

PART-II Obstetric Information

| S.No | Question | Respondents possible answer | Code | Skip |
| --- | --- | --- | --- | --- |
| 201 | What is the number of births you gave up to now(include if there is still birth or died soon) | ----------------- |  |  |
| 202 | At what age was your first pregnancy | -------------------years |  |  |
| 203 | What outcomes did you face during your pregnancy and child birth? (more than one answer is possible) | 1-Abortion  2-Live birth  3-Still birth  4-Others,specify----------- |  |  |
| 204 | Are you pregnant now | 1-Yes 2-No |  |  |

PART-III-Women’s knowledge of obstetric danger sign

Instruction - Put “X” mark on free columns after the response of respondents, i.e. Weather answering the question spontaneous or after explanation

| S.No | Variables | Respondents possible answer | Spontaneous | After explanation |
| --- | --- | --- | --- | --- |
| 301 | Do you know any danger sign throughout pregnancy, delivery and soon after birth? | 1-Yes  2-No |  |  |
| 302 | If yes, from where did you get the information? | 1-Health workers  2-HEW  3-Community health Promoters  4-Radio  5-Television  6-My neighbors  7-Read pamphlets/books  8-Others,specify--------------- |  |  |
| 303 | Do You think that these danger signs threaten the life of the women | 1-Yes 2-No |  |  |
| 304 | What danger signs are there during pregnancy? | 1-Severe vaginal bleeding  2-Swollen hand and face  3-Blured vision  4-head ache  5-decrese fetal movement  6-other specify  7-I don’ know |  |  |
| 305 | What danger signs are there during child birth? | 1-Severe bleeding  2-Prolonged labour  3-Convlusion  4-Retained placenta  5-other specify  6-I don ‘know |  |  |
| 306 | What are the danger signs that occur soon after birth? | 1-Severe vaginal bleeding  2-Foul smelling of vaginal discharge  3-High fever  4-Blurred vision  5-other specify  6- I don ‘know |  |  |
| 307 | Do you think ANC is useful? | 1-Yes 2-No |  |  |
| 308 | Do you think that preparing for delivery is important? | 1-Yes 2-No |  |  |
| 309 | What are BPCR | 1-identify place of delivery  2-Cloth for newborn  3-Identified helper  4-save money  5-arrange transport  6-prepare blood donor  7-prepare different items  8-knowing danger sign |  |  |
| 310 | Do you think that plan for place of delivery is important? | 1-Yes 2-No |  |  |
| 311 | Do you think that delivering at facility is useful? | 1-Yes 2-No |  |  |
| 312 | Is plan to have skilled attendant at delivery useful? | 1-Yes 2-No |  |  |

PART-Iv Information on service delivery (practice)

| 401 | Did you attend ANC before? | 1-yes 2-No |  |  |
| --- | --- | --- | --- | --- |
| 402 | Do you attend ANC during your last pregnancy? | 1-Yes 2-No |  |  |
| 403 | Who attended your ANC visit? | 1-Skilled health professional (Midwife, Nurse & Doctor)  2-HEW  3-TBA  4-Others,specify---------- |  |  |
| 404 | At what week/month did you start your ANC? | ----------------week/month |  |  |
| 405 | How many times you attended ANC in your last pregnancy? | 1-Once  2-Twice  3-Three times  4-Four and above |  |  |
| 406 | Did you make any preparation in your last pregnancy? | 1-Yes 2-No |  |  |
| 407 | What preparations did you make? | 1-Grain for porridge  2-Cloth for newborn  3-Identified helper  4-Others,specify--------- |  |  |
| 408 | Did you plan for place of delivery? | 1-Yes 2-No |  |  |
| 409 | Where do you plan place of delivery?(only for pregnant | 1-Home  2-Health post  3-Health center  4-Hospital  5-Others,specify--------- |  |  |
| 410 | Where were your last deliveries? | 1-Home  2-Health post  3-Health center  4-Hospital  5-Others, specify---------- |  |  |
| 411 | Why do you prefer to deliver at home?(Possible to answer more than one answer) | 1-Need to be with relatives  2-Prefer home ceremony after delivery  3-B/c of the cost is cheap  4-Presence of TBAs  5-My husband accepts it  6-My previous home deliveries were normal  7- Lack of transport  8-The health facility is too far  9-Health care providers approach are not good  10-No female provider  11-Lack of accompanying family members  12-Others,specify |  |  |
| 412 | Why do you prefer to deliver at health facility? | 1-The facility is near to me  2-Gave better service  3-I had better out come before  4-Health workers advice  5-Difficulty of labour  6-I had problem with previous home deliveries  7-Others,specify------------ |  |  |
| 413 | In what way did you give birth before? | 1-Spontaneous vaginal delivery  2-Instrumental delivery  3-Cesarean section  4-I didn’t remember  5-Others,specify---------------- |  |  |
| 414 | Have you given birth at health facility? | 1-Yes 2-No |  |  |
| 415 | If Yes, how many births have you given? | ------------- |  |  |
| 416 | Who attended your delivery before? | 1-Doctor  2-Nurse  3-HEW  4-TBAs  5-Others ,specify |  |  |
| 417 | What was the outcome of your last delivery? | 1-Live birth  2-Live birth but died soon  3-Still birth  4-Others,specify-------------- |  |  |
| 418 | Did you save money for your previous delivery? | 1-Yes 2-No |  |  |
| 419 | Did you plan for transport for emergency at delivery? | 1-Yes 2-No |  |  |
| 420 | So what was your transport? | 1-On foot  2-On hoarse back  3-Carried by people  4-Seeking ambulance  5-Others,specify |  |  |
| 421 | Did you prepare blood donor | 1-Yes 2-No |  |  |
| 422 | Do you think it is important | 1-Yes 2-No |  |  |
| 423 | Have you been encountered with any problem during pregnancy, delivery and after birth | 1-YES 2-No |  |  |
| 424 | If yes, what was the problem | 1-Excessive vaginal bleeding  2-prolonged labour(>12 hr0  3-Retained placenta(>1 hr)  4-Inability to control urine/faces/both  5-Mal presentation  6-Fetal death  7-Early rapture of membrane  8-Fver and offensive vaginal discharge  9-Loss of consciousness  10-Others ,specify |  |  |
| 425 | Were you referred to next the facility? | 1-Yes 2-No |  |  |
| 426 | If yes, who went with you there? | 1-Husband  2-Relatives  3-Community emergency committee  4-With health care provider  5-Others ,specify |  |  |
| 427 | What order was your last birth? | 1-First 4- Four and above  2-Second 3-Third |  |  |

##

## 10.3. Annex III: Amharic consent form

mGl´ ymréA³ yfšDmeyq#¶ wrqT

ደሕናአደሩ/ደህናዋሉ/ደህናአመሹ፣ሰሜነኢማየሱፍይባላል (መረጃሰብሳቢነኝ) ym½uƒTkaë^Sab²uMl^N×l_Jነዉ፡ yሚድዋይፈሪtìÞnI>¥¥ከወሊድናከወሊድበኋላለሚከሰቱዓደገኛምልክቶችዕንዲሁምየገጠርዕናቶችለወሊድበቂዝግጅትያደርጋሉወይስዓያደርጉምበሚልጽንሰሀሳብዙሪያበመዉለድዕድሜክልልዉስጥባሉሴቶችላይጥናትለማካሄድነዉ፡፡ይህነንጥናትለማካሄድየማህበራዊናስነ-ህዝባዊ፣ከርግዝናናወሊድጋርታይዘዉለሚፈጠሩዓደገኛምልክቶች፣ለወሊድየሚደረግበቂዝግጅትዕናየጤናዓገልግሎትዓጠቃቀምንበተመለከተመረጃዓሰባስባለሁ፡፡ ARS¿ lE³t>tmRe§L¥ዓኁንፈቃደኛከሆኑጥቂትቃለመጠይቆችንከርሶጋርማድረግዕፈልጋለሁ፡፡ለተጠየቁትጥያቄዎችበሙሉወይምበከፊልዕንዲሁምያለመመስመብቶየተጠበቀነዉ፡፡ጥናቱንማቆረጥበእርስዎላይምንምተፅኖአያመጣም፡፡ለቃለመጠይቁየሚያስፈልገዉጊዜ 30 ደቂቃይሆናል፡፡ ከዕርሶየሚሰበሰበዉመረጃለዚህጥናትብቻየሚዉልይሆናል፡፡በዚህጥናትየምሰጡትመረጃበሚስጥርነዉሚያዘዉስMaYeqSMበተለየኮድነዉì@mzgbˆ ¥¥ byT¼ˆM s¹T ìB™R¶ kflgymeyQmBTal¿T¥¥

ቃለመጠይቁንመቀጠልዕችላለሁ/ ዓዎ/ዓይደለም

በምላሹመሰረትቃለመጠይቁንይቀጥሉ

አዎ--------------ከሆነወደሚቀጥለዉጥያቄእለፍ/ፊ/

የለም ------------ከሆነጥያቄዉንአቆርጥ/ጭ/

የጠያቂዉሥም------------------------------------------------------ፊርማ--------------------ቀን---------------Amharic Version questionnaire በዓማርኛየተዘጋጀቃለመጠይቕ

የሱፐርቫይዘሩስም------------------------------------------------- ፊርማ-----------ቀን-------------

የጥያቄኮድ----------------------- ቀበሌ-------------- ጎጥ------------- የቤትቁጥር-------------

ማሳሰቢያ፡ምርጫያላቸዉንመልሶችንይክበቡዝርዝርየሚያስፈልጋቸዉንበክፍትቦታላይይጻፉ

| ተራ.ቁ | ጥያቄዎች | ዓማራጭመልሶች | ኮድ | ወደሌላጥያቄ |
| --- | --- | --- | --- | --- |
| 101 | ዕድሜስንትዓመትነዉ | -------------------------- |  |  |
| 102 | የትምህርትደረጃስንትነዉ | 1-ዓልተማርኩም 2-ማንበብናመጻፍ  3-ከ1ኛ-8ኛክፍል 4-2ኛደረጃናከዚያበላይ |  |  |
| 103 | ስራዎምንድነዉ | 1-የቤትዕመቤት 2-ዓርሶዓደር  3-የቤትሰራተኛ 4-የመንግስትስራተቀጣሪ  5-የግልመስሪያቤትተቀጣሪ 6-ነጋዴ  7-ሌላካለይጥቀሱ---------------------------------------- |  |  |
| 104 | በወርስንትብርያገኛሉ | ----------------------ብር |  |  |
| 105 | የጋብቻሁኔታዎዕንዴትነዉ | 1- ያገባች 2-ያላገባች  3-የተፋታች 4-ባልየሞተባት  5-ዓግብታየማታዉቅ 6-ሌላካለይጥቀሱ--------- |  |  |
| 106 | መጀመሪያሲያገቡዕድሜዎስንትነበር | -------------ዓመት |  |  |
| 107 | የባለቤትዎየትምህርትደረጃምንይመስላል(ላገቡብቻየሚጠየቅ) | 1-ዓልተማርም 2-ማንበብናመጻፍ  3-ከ1ኛ-8ኛክፍል 4-2ኛደረጃናከዚያበላይ  5-ሌላካለይጥቀሱ---------------------------------------- |  |  |
| 108 | የባለቤትዎስራምንድነዉ | 1-ዓርሶዓደር 2-የመንግስትሰራተኛ  3-የግላተቀጣሪ 4-ነጋዴ  5-የቀነጉልበትሰራተኛ 6-ሌላካለይጥቀሱ--------------- |  |  |
| 109 | ባለቤትዎከሚሰራዉስራምንያህልገቢያገኛል | -----------------------ብር  ---------------------------በዓይነት |  |  |
| 110 | በዓንድቤትዉስጥስንትሆናቹትኖራላቹ | --------------------- |  |  |
| 111 | በቤተሰቡዉስጥዉሳኔሰጪማነዉ | 1-ዕኔራሴ 2-ባለቤቴ  3-ሁለታችንም 4-ሌላካለይጥቀሱ---------------- |  |  |

## .4. Annex IV: Amharic questioner

ክፍልዓንድ፡ማህበራዊናስነህዝባዊመረጃዎች

ክፍልሁለት፡ከዕርግዝናከወሊድናከወሊድበኋላሁኔታዎችጋርየተያያዘጥያቄ

| ተራ.ቁ | ጥያቄዎች | ዓማራጭመልሶች | ኮድ | ወደሌላጥያቄ |
| --- | --- | --- | --- | --- |
| 201 | ዕስካሁንስንትግዜወልደዋል(በህይወትየተወለደ፣ሙቶየተወለደናወዲያዉዕንደተወለደየሞተ) | ----------------------ግዜ |  |  |
| 202 | ለመጀመሪያግዜሲያረግዙዕድሜዎስንትነበር | ------------------------ዓመት |  |  |
| 203 | በዕርግዝናናበወሊድግዜምንዓይነትችግርዓጋጥሞትነበር | 1-ዉርጃ2-ህይወትያለዉልጅመዉለድ  3-የሞተልጅመዉለድ 4-ሌላካለይጠቀስ------------ |  |  |
| 204 | ዓኁንነፍሰጡርኖት | 1-ዓዎ 2-ዓይደለም |  |  |

ክፍል3- ከርግዝና፣ከወሊድዕና፣ ከወሊድበኋላለሚከሰቱዓደገኛየጤናችገሮችየግንዛቤቃለመጠይቅማሳሰቢያ - በተቀመጠዉከፍትቦታላይ የ ዔክስ(x) ምልክትያስቀምጡሲያስቀምጡምምላሹንየሰጡትወዲያዉከሆነወዲያዉኑየሚለዉከፍትቦታላይከማብራሪያበኋላከሆነደግሞበተቀመጠዉክፍትቦታላይያስቀምጡ

| ተራ.ቁ | ጥያቁዎች | ዓማራጭመልሶች | ወዲያዉኑ | ከማብራሪያበጓላ |
| --- | --- | --- | --- | --- |
| 301 | በርግዝነ፣በወሊድናከወሊድበኋላያሉትንዓደገኛምልክቶችዕንዳሉያዉቃሉ | 1-ዓዎ 2-ዓይደለም |  |  |
| 302 | ከሆነከየትሰሙ | 1-ከጤና ባለሞያ 2-ከጤና ዔከስቴንሽን  3-ከበጎ ጤናመልክተኛ 4-ከሬድዮ  5-ከቴሌቪዢን 6-ጎሮቤቶቼ ነግለዉኝ  7-ከመጻህፍትና በራሪወረቀቶች 8-ሌላ ካለ------------ |  |  |
| 303 | ዕነዚህዓደገኛምልክቶችለሞትየሚዳርጉ መ4ሆኑን ያዉቃሉ | 1-ዓዎ 2-ዓይደለም |  |  |
| 304 | በርግዝናግዜየሚከሰቱዓደገኛምልክቶችንምንድናቸዉ | 1-ብዙ ደምመፍሰስ2-የፊትና ዕጅላይዕበጠት  3-የዓይን ብዠታ4-የዕንሽርት ዉሀዉፈሶ  5-የልጅ እንቅስቃሴመቀነስ 6-ራስ ምታ  7-ዓላዉቅም 8-ሌላካለይጥቀሱ |  |  |
| 305 | በወሊድግዜየሚከሰቱዓደገኛምልክቶችምንድናቸዉ | 1-ከማህጸን ብዙደምመፍሰስ  2- ለረጅምግዜየቆየምጥ  3-ማንዘፍዘፍ  4--ዕንግዴልጅሳይወጣመቆየት  5-ሌላ ካለ---------  6-ዓላዉቅም |  |  |
| 306 | ከወሊድበኋላየሚከሰቱዓደገኛምልክቶችምንድናቸዉ | 1--ከማህጸንብዙደምመፍሰስ  2-መጥፎ ጠረንያለዉየማህጸንፈሳሽ  3-ከፍተኛ ትኩሳት  4-የዓይን ብዠታ  5-ሌላ ካለ---------  6-ዓላዉቅም |  |  |
| 307 | በዕርግዝናግዜየሚደረግምርመራያስፈልጋልብለዉያስባሉ | 1-ዓዎ 2-ዓይደለም |  |  |
| 308 | ለወሊድበቂዝግጅትማድረግያስፈግጋልብለዉያስባሉ | 1-ዓዎ 2-ዓይደለም |  |  |
| 309 | የሚወልዱበትንቦታማቀድይጠቅማልብለዉያስባሉ | 1-ዓዎ 2-ዓይደለም |  |  |
| 310 | በጤናተቋምመዉለድይጠቅማልብለዉያስባሉ | 1-ዓዎ 2-ዓይደለም |  |  |
| 311 | በጤናባለሞያዕንዲያግዞትማቀድይጠቅማልብለዉያስባሉ | 1-ዓዎ 2-ዓይደለም |  |  |

ክፍል4፡ የጤናዓገልግሎትዓጠቃቀምንበተመለከተየሚጠየቅቃለመጠይቅ

| 401 | ከዚህበፊትበነበሩትየዕርገዝናጊዜዎበቂየዕርግዝናምርመራዓድርገዉነበር | 1-ዓዎ 2-ዓይደለም |  |  |
| --- | --- | --- | --- | --- |
| 402 | ባሁኑዕርግዝናዎየቅድመወሊድክትትልዓድርገዉነበር | 1-ዓዎ 2-ዓይደለም |  |  |
| 403 | በስንተኛዉሳምንት/ወርክትትሉንዓደረጉ | ----------------ወር/ሳምንት |  |  |
| 404 | የርግዝናምርመራዉንየከናወነዉማነዉ | 1-ዶክተር 2-አዋላጅ ነርስ  3-ጤና ዔክስቴንሽን4-ልምድ ዓዋላጅ  5-ሌላ ካለይጥቀሱ-------------------------------- |  |  |
| 405 | በመጨረሻዉዕርግዝናስንትግዜክትትልዓደረጉ | 1-ዓንድ ግዜ 2-ሁለት ግዜ  3-ሶስት ግዜ 4-ዓራትና ከዚያበላይ |  |  |
| 406 | ለወሊድየሚያስፈልጉዝግጅቶችንዓድርገዉነበር | 1-ዓዎ 2-ዓይደለም |  |  |
| 407 | መልሶዓዎከሆነምንምንዝግጅትዓደረጉ | 1-የገንፎ ዕህል 2-የልጅ ልብስ  3-የሚያርስ ሰዉ 4-ሌላ ካለየጥቀሱ----- |  |  |
| 408 | የትዕንደሚወልዱዓቅደዉነበር | 1-ዓዎ 2-ዓይደለም |  |  |
| 409 | የትነበርያቀዱት(ለነፍሰጡርብቻየሚጠየቅ) | 1-ቤት 2-ጤና ኬላ  3-ጤና ጣቢያ 4-ሆስፒታል  5-ሌላ ካለይጥቀሱ------------------------- |  |  |
| 410 | ባለፈዉየትነበርየወለድሽዉ | 1-ቤት 2--ጤናኬላ  3-ጤና ጣቢያ 4-ሆስፒታል  5-ሌላ ካለይጠቀሱ--------- |  |  |
| 411 | ቤትዉስጥመዉለድለምንመረጡ(ካንድበላይመልስመስጠትይቻላል) | 1-ከቤተሰቤ ጋርመሆንስለፈለኩኝ  2-ከወለድኩኝ በኋላበቤትዉስጥያለዉንዝግጅትስለፈለኩኝ  3-ምንም የማያስከፍልስለሆነ  4-የልምድ ዓዋላጅስላለ  5-ባለቤቴ ሰለተቀበለዉ  6-ከዚህ በፊትቤትወላጄምንምችግርስላልገጠመኝ  7-ትራንስፖርት ስለሌለ  8-የጤና ተቋሙበጣምስለሚርቅ  9-ጤና ባለሞያዎቹያላቸዉዓቀራረብጥሩስላልሆነ 10-ሴት የጤናባለሞያስለሌለ  11-ዓብሮኝ የሚሄድቤተሰብስለሌለኝ  12-ሌላ ካለይጠቀስ----------------------------- |  |  |
| 412 | በጤናተቋምመዉለድለምንመረጡ | 1-ጤና ድርጅቱቅርብስለሆነ  2-የተሻለ ዓገልግሎትስለሚሰጥ  3-ባለፈዉ በጤናድርጅትበጥሩሁኔታስለወለድኩኝ  4-የጤና ባለሞያዎቹምክር  5-ምጡ በጣምስለጠናብኝ  6-ባለፈዉ በቤትዉስጥስወልድችግርስላጋጠመኝ  7-ሌላካለ ይጠቀስ------------------------------- |  |  |
| 413 | ባለፈዉበምንዓይነትሁኔታነዉየወለዱት | 1-በራሴ ዓምጬወለድኩ 2-በመሳሪያ ታግዤ  3-የማህጸን ቀዶጥገናተደርጎልኝ 4-ዓላስታዉስም  5-ሌላ ካለይጥቀሱ-------------------------------- |  |  |
| 414 | ከዚህበፊትበጤናተቋምወልደዉያዉቃሉ | 1-ዓዎ 2-ዓይደለም |  |  |
| 415 | መልሶዓዎከሆነስንትግዜበጤናተቋምወልደዋል | --------------ግዜ |  |  |
| 416 | ማንነበርያዋለደሽ | 1-ዶክተር 2 ነርስ  3-ጤና ዔከስቴንሽን4-ልመወድ ዓዋላጅ  5-ዓዋላጅ ነርስ 6-ሌላ ካለ |  |  |
| 417 | ህጻኑሲወለድየመጨረሻሁኔታዉምንይመስላል | 1-ህይወት ያለዉ 2-በህይወት ተወልዶወዲያዉየሞተ  3-ሞቶ የተወለደ 4-ሌላ ካለ ------------------- |  |  |
| 418 | ከርግዝናናወሊድጋርተያይዞለሚፈጠርችግርየሚሆንገንዘብለማስቀመጥዓቅደዉነበር | 1-ዓዎ 2-ዓይደለም |  |  |
| 419 | ድንገትለሚፈጠሩዓስቸጋሪሁኔታዎመጓጓዣዓዘጋጅተዉነበር | 1-ዓዎ 2-ዓይደለም |  |  |
| 420 | ከሆነያዘጋጁትመጓጓዣምንነበር | 1-በዕግር 2-በፈረስ/በበቅሎ  3-ሰዎች በቃሌዛተሸክመዉኝ  4-ዓመቡላንስዕጠብቃለሁኝ  5-ሌላ ካለ--------------------------- |  |  |
| 421 | ከላይየተጠቀሱትድንገተኛችግሮችቢከሰቱደምየሚሰጥሰዉዓቅደዉነበር | 1-ዓዎ 2-ዓይደለም |  |  |
| 422 | ይህንንማቀድጠቃሚነዉብለዉያስባሉ | 1-ዓዎ 2-ዓይደለም |  |  |
| 423 | ባለፈዉሲወልዱከርግዝናናወሊድጋርተያይዞያጋጠሞትችግርነበር | 1-ዓዎ 2-ዓይደለም |  |  |
| 424 | መልሶዓዎከሆነምንዓይነትችግርነዉያጋጠሞት | 1-ብዙ ደምመፍሰስ  2-ለረጅም ግዜየቆየምጥ  3-ዕንግዴ ልጅሳይወጣቆይቷል  4-ሽንትና ሰገራመቆጣጠርዓቅቶኝነበር  5-የጽንሱ ዓቀማመጥያልተስተካከለነበር  6-ጽንሱ በሁዴጸፍቶነበር  7-የዕንሽርት ዉሀዉፈሶቆይቶነበር  8-ትኩሳትና መጥፎሽታያለዉየማህጸንፈሳሽ  9-ራስን መሳት  10-ሌላ ካለ ------------------------ |  |  |
| 425 | ለነዚህችግሮችወደጤናተቋምሄደዉነበር | 1-ዓዎ 2-ዓይደለም |  |  |
| 426 | ከሆነዓብሮሽየሄደዉማነዉ | 1-ባለቤቴ 3-የዕድር ዓባላት  2-ዘመዶቼ 4-ጤና ባለሞያ  5-ሌላ ካለ---------- |  |  |
| 427 | ባለፈዉየተወለደዉልጅስንተኛልጆነዉ | 1-ዓንደኛ 3-ሶስተኛ  2-ሁለተኛ 4-ዓራተኛ |  |  |
